# Beyond Silence: A Scoping Review of Provided Support for Grieving Children With Intellectual Disabilities or Autism Spectrum Disorder

**DOI:** 10.1177/00302228231226343

**Published:** 2024-01-04

**Authors:** Maria Bonin, Lilly Augustine, Qi Meng

**Affiliations:** 1 School of Education and Communication, 318951 Jönköping University, Jönköping, Sweden

**Keywords:** grief support, intervention, children, autism spectrum disorder, intellectual disabilities

## Abstract

Children with intellectual disabilities (ID) or autism spectrum disorder (ASD) are considered unable to grieve or understand the concept of death and might not receive grief support after the death of a beloved person; hence, they are at risk of developing complicated grief. This scoping review identified existing grief support for children with ID or ASD. Searching seven databases yielded 514 records; six studies met the predefined inclusion criteria. The six studies identified grief support, including discussions, participation in death rituals, family support, stories, and professional interventions. The support could be organized into three levels, micro, meso, and exo, overlooking the macro level completely, indicating that grief support for these children tends to be irregular and inconsistent.

## Introduction

People with intellectual disabilities (ID) or autism spectrum disorder (ASD) experience grief when they lose a loved one (Brickell & Munir, 2008; Dodd et al., 2005; McRitchie et al., 2014). However, they often face the prejudice that they are incapable of grieving, especially if they have difficulties expressing themselves. As a result, they experience exclusions from grieving processes and grief support (Hermosilla & Robaina, 2021).

Children with ID are considered unable to understand the meaning of death’s finality due to intellectual and adaptive behavior deficits (Dodd et al., 2005). Children’s developmental stage influences their understanding of death and grief response (Kronaizl, 2019). Grieving after losing a loved one, however, is a universal experience that children of all ages can experience, although the manifestation and expression vary (D’Antonio, 2011). Thus, experiencing that a loved one is gone and grieving does not necessarily require a complete comprehension of death. A similar misunderstanding exists regarding children with ASD (Sormanti & Ballan, 2011) based on their limitations in social communication and interactions. Furthermore, a misguided intention of protecting children from strong negative emotions leads to limited participation in grieving processes and rituals (Dodd et al., 2005).

Children with ID or ASD display a broad range of grief reactions, and these strong emotions negatively impact their everyday functioning (Gaines, 2022). The spectrum of social-emotional grief reactions includes crying, hitting, overstimulation, and losing interest in daily or favorite activities. Moreover, new behaviors could arise, indicating difficulties coping, for example, aggressive or repetitive behavior, compulsivity, withdrawal, or sleeping difficulties (Sormanti & Ballan, 2011). Arguably, grief can be complex for some children, especially when communication is challenging (Clute, 2010). Grief reactions of those children may be misinterpreted as challenging behavior caused by the disability (Dodd et al., 2005; MacHale & Carey, 2002), leading to the need for support being overlooked. Deficits in communication, understanding, social skills, and interpersonal relationships make them vulnerable in challenging times after the loss (Sormanti & Ballan, 2011).

Creating a supportive environment and providing support for grieving children is vital. Both children with ASD and ID are at risk of being overlooked after the death of a beloved person and not receiving the needed support (Hermosilla & Robaina, 2021). Excluding these children from grieving hinders healthy grief reactions and can have negative consequences for their mental health (Read & Elliott, 2007; Stroebe et al., 2007). Children with ID or ASD are at risk of developing complicated grief and adverse grief outcomes (Dodd et al., 2008; Hermosilla & Robaina, 2021; Sormanti & Ballan, 2011). Thompson and Doka (2017) described complicated or disenfranchised grief as a person’s experience that others do not acknowledge or recognize. It pertains to an individual’s inability to mourn or receive societal support during their grieving experience.

A supportive environment can positively impact the grieving process; vulnerable individuals need a supportive environment to cope with the loss (Aoun et al., 2015; Dyregrov, 2008). Some children, however, might require professional grief support and interventions (Dyregrov & Dyregrov, 2008). Appropriate support and evidence-based interventions are necessary to support those vulnerable children and minimize the risk of developing mental disorders. Adapted grief support for children with ID or ASD is crucial to ensure the best possible care for this vulnerable group (Duc et al., 2017).

Research about grief support for children with ID and children with ASD is scarce (Sormanti & Ballan, 2011). Most studies about grief support focus either on adults or children without disabilities. Existing research on grief in people with ID or ASD mainly focuses on adults (Clements et al., 2004; Lord et al., 2017; Young et al., 2017). Gaines (2022) recently published a literature review about the grief experiences of children with developmental disabilities, including children with ID and ASD. However, the article focuses on grief experiences and expressions without the provided support. Gilrane-McGarry and Taggart (2007) emphasized the need for integrating continuous grief counseling into the everyday life of grieving individuals with cognitive disabilities. This approach is reflected in the bereavement support model by Read and Elliott (2007), which includes various support strategies on different environmental levels (micro, meso, exo, and macro level), from the family of the child (micro) to the policy level nationally (macro). The model contains four components of support (education, participation, facilitation, and intervention). The integrated approach of the Read and Elliott (2007) bereavement support model is adopted in this study for children with ID or ASD to analyze and discuss components of grief support for those children. Included studies contain support from the social network, as well as professional grief interventions.

This scoping review aims to identify provided grief support for children with ID or ASD after a beloved person’s death and then analyze provided support depending on the environmental level and component based on the bereavement support model by Read and Elliott (2007).

## Method

### Search Strategy

In November 2022, when searching for literature, the search strings created were based on the Population, Intervention, and Outcome of interest (see Table 1). All relevant terms concerning, for example, Population were combined using the Boolean ‘OR,’ and all three areas were connected using the Boolean ‘AND.’ As grief support can relate to psychological research, social science, and educational research, databases indexing publications in these areas were selected: Seven in total (Education Collection, PsychINFO, ProQuest Central, Scopus, Social Science Premium Collection, Sociology Collection, and Web of Science).

**Table 1.** Search Strings Related to Population, Intervention and Outcome.

Area	Search String
Population	“Intellectual disabilit*” OR “mental retardation” OR “intellectual impairments” OR “mentally disabled” OR disabilit* OR “special needs” OR ASD OR Autis* AND child* OR adolescen* OR youth OR teenager
Intervention	Grief OR bereave* OR mourn* OR death OR died
Outcome	Support OR intervention OR program OR counselling OR therap*

In ProQuest Central, a simplified search was needed, title(Grief) AND noft(autis* OR “intellectual disabilities” OR “intellectual disability”) AND noft(child*), as it generated additional articles. Also, relevant literature was screened to identify potential studies via hand searching.

### Selection Process

**Figure 1.**
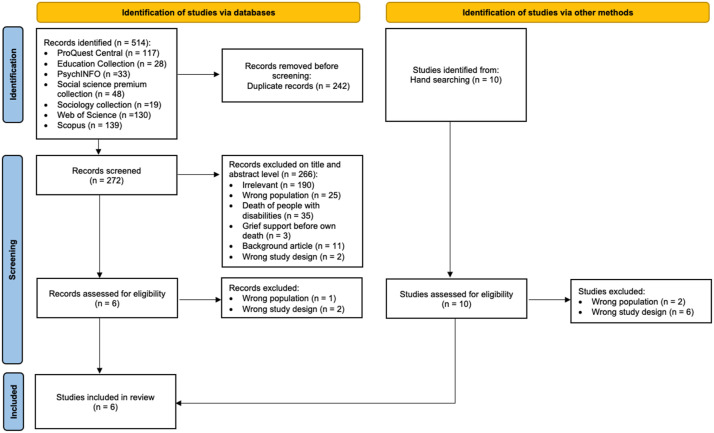
PRISMA flowchart.

Articles were selected by predefined inclusion and exclusion criteria (see Table 2), focusing on grief support for children with ID or ASD between 0 and 18 years. The target population was limited to children who had lost a beloved person directly involved in their daily lives. The initial search identified 514 articles, including 242 duplicates, leading to a title and abstract screening of 272 articles, excluding 266, leaving six articles for full-text screening. Eventually, three articles were excluded due to the wrong population or study design, and three were included. Three additional studies were identified by hand-searching literature relevant to the topic, which resulted in six articles in total in this scoping review. See the PRISMA flowchart (Page et al., 2021), which identifies the different steps in the screening process (Figure 1).

**Table 2.** Inclusion and Exclusion Criteria.

	Inclusion Criteria	Exclusion Criteria
Population	Children with ID or ASD who encountered the death of a loved one (family member, caregiver, teacher, friend). This person had to have been involved in the children’s everyday life.	Children with ID or ASD facing their death, adults with ID or ASD, parents of children with ID and ASD.
	Children with diagnosed ID or ASD, 0–18 years	If more than one child with ID or ASD is older than 18 years
Intervention	Grief support, counseling, or interventions that aims to help grieving children with ID or ASD as the focus or subtopic	Medication, hospitalization, hospice or palliative care for children with ID or ASD
Outcome	Components of grief support	Identification of risk factors
Study design	Quantitative and qualitative studies, mixed methods, case studies	Literature reviews, systematic reviews, theoretical papers
Publication type	Peer-reviewed articles, doctoral theses, publication 2000–2022, in English or German	Books, book chapters, student theses, conference proceedings

*Note.* ID = intellectual disability; ASD = autism spectrum disorder.

### Quality Assessment

The Critical Appraisal Skills Programme (2022, November 26) checklist for qualitative studies was used to assess the quality of the remaining studies. Five studies had high quality (Ducy & Stough, 2018; Haider & Zaman, 2022; Johnson, 2016; Lewis, 2019; McClean & Guerin, 2019) and one low quality (Zakreski, 2017). The low quality of one article was mainly due to the lack of information regarding participant recruitment and ethical considerations. Furthermore, the author’s and participant’s relationship was not considered, and the article’s aim was unclear.

### Data Extraction and Analysis

MB extracted the data utilizing an extraction protocol developed beforehand. The extracted data comprised information about the study design, participants, type, content of grief support, and the study’s outcome, if reported. Based on Read and Elliott (2007), a focus is on the type of support, such as education, participation, facilitation, or intervention, as well as what environmental level is used (micro, meso, exo, or macro).

## Results

### Overview of Selected Studies

Six studies were included in this literature review. All studies had a qualitative study design and focused on different forms of support for children with ID or ASD. The studies were peer-reviewed and published from 2016 to 2022. Each study received an identification number (IN) to simplify the references (see Table 3).

**Table 3.** Overview of Included Studies.

IN	Author (Year)	Participants	Children’s Age	Country	Aim of the Study	Study Design	Quality Assessment
1	Lewis (2019)	Teachers of children with ASD (*n* = 8), parents of children with ASD (*n* = 5)	Kindergarten to 3^rd^ grade	USA	Identifying what children’s books are sufficient for supporting children with ASD through grief.	Qualitative study, focus groups	+
2	Haider and Zaman (2022)	Adolescents with ID (*n* = 7)	10–19 years	Pakistan	Exploring how adolescents with ID understand, process, express, and manage grief.	Qualitative study, semi-structured interviews	+
3	Zakreski (2017)	Adolescent with ASD (*n* = 1)	15 years	USA	Exploring the impact of the loss on the adolescent and supporting them in exploring and expressing emotions.	Qualitative study, case study	−
4	Ducy and Stough (2018)	Special educators (*n* = 5)	Elementary school students	USA	Exploring teachers’ perceptions of grief in children with ID and the teachers’ responses.	Qualitative study, semi-structured interviews	+
5	McClean and Guerin (2019)	Psychologists (*n* = 12)	No information	Ireland	Examining grief experiences among children with ID, reported by psychologists.	Qualitative study, semi-structured interviews	+
6	Johnson (2016)	Adolescents with ASD (*n* = 4)	12–16 years	USA	Investigating the experiences of adolescents with ASD who engaged in grief therapy using video games.	Qualitative study, collective case study	+

*Note.* IN = Identification number; ID = Intellectual disability; ASD = Autism spectrum disorder; Quality Assessment (+ = high quality; 0 = moderate quality; − = low quality).

Four studies were peer-reviewed papers (2, 3, 4, 5), and two were PhD theses (1, 6). Out of the six studies, four were set in the USA (1, 3, 4, 6), one in Ireland (5) and one in Pakistan (2). Two studies focused on elementary school children (1, 4), and one did not include information about the children’s age (5). Only three studies reported the children’s specific age (2, 3, 6). Two of these focused on adolescents with ASD (3, 6), and one on adolescents with ID (2); of the other three, one focused on ASD (1) and two on ID (4, 5). In one study, children with ASD were mentioned while focusing on children with ID (5). Due to inconsistencies in age, the term children will also include adolescents.

Three studies comprised support from social networks like school and family (1, 2, 4), and three focused on professional interventions (3, 5, 6). The data was collected by involving either the children themselves (2, 3, 6), parents (1), teachers (1, 4), or psychologists (5). Methods used for data collection were focus group discussions (1), semi-structured interviews (2, 4, 5), and case studies (3, 6) (see Table 3).

### Support for Grieving Children with ID or ASD

How to provide support for grieving children with ASD or ID was explicitly mentioned in five of the six studies (1, 3, 4, 5, 6). In one study, participants described what they experienced as helping manage the loss without a detailed description of the support they received (2). Another study contained suggestions from parents and teachers of how helpful the support might be (1). Two studies included detailed descriptions of professional support (3, 6), and two studies comprised the experiences of support providers (4, 5) (see Table 4).

**Table 4.** Identified Grief Support.

Content IN:	1	2	3^*^	4	5^*^	6^*^	Sum
Death-related books and stories	X			X	X		3
Preparation for death rituals, e.g., funerals	X			X	X		3
Participation in rituals, e.g., funerals		X				X	2
Crafts to remember and honor the deceased person				X			1
Prayers and religious beliefs		X					1
Discussions about death and dying	X		X	X	X	X	5
Education about death, dying, and emotions	X		X	X	X	X	5
Advising the family on how to respond to children’s grief (indirect help)			X	X	X		3
Connecting the family to other resources (indirect help)	X			X	X		3
Video games						X	1
Complicated grief treatment			X				1

*Note.* *Studies with professional interventions; IN: Studies’ identification number.

When analyzing the type of support in these five studies, we found that most studies addressed support related to talking about death or educating about death and emotions. These types of support are aimed at helping children understand the situation, their feelings, and other people’s reactions to the death of a beloved person. Other common types of support related to preparing children for death rituals included explanations of what to expect on occasions like funerals. As visualized in Table four, only two studies reported the participation of children with ID or ASD in death rituals as a way of support.

Books and stories were often used to explain emotions, learn coping strategies, and normalize the grief experience. Stories were also employed as video games to facilitate discussions around death and emotions. Additionally, books were used to prepare for death rituals and education. Especially for children with ASD, they are required to be written in straightforward and clear language. One study considered religious ideology and prayers helpful. Cards were created to honor and remember the beloved person and support the children in grieving. Indirect support was provided in four studies by connecting the family or parents to other resources and enabling parents to support their child. In two studies, the children’s grief response overwhelmed the parents, which resulted in them seeking additional help for the grieving children.

### Professional Support for Children with ID or ASD

Three studies (3, 5, 6) contained professional grief interventions conducted by psychologists or a counselor with a background in psychology (see Table 4). A component of professional interventions was the indirect support for parents to help them normalize the grief experience for the family and encourage them to include children in death rituals and grieving processes.

The supportive stories in books and video games incorporated a story filled with topics of death, loss, grief, and the strength of coping with it. Video games helped children recognize and understand emotions and ways of dealing with them. Children who communicated less verbally could explore grief-related feelings through stories and talking about someone else’s experiences. The participants avoided reflections on their grief, and no participant expressed a personal meaning of grief; they mostly referred to the story’s character. After the grief counseling, the participants’ caregivers recognized some minor changes in the grieving children. One participant, for example, showed empathy for the mother’s sadness and shared feelings with her. However, whether it was due to the intervention or personal development is unclear.

Another study focused on treating complicated grief symptoms in a participant with ASD. That intervention included the complicated grief treatment (CGT) approach, containing aspects of motivational interviewing, interpersonal therapy, and cognitive-behavioral therapy. It aimed to explore the participant’s emotions and help them to express them to deal with the impact of the loss. Although the treatment was recognized as being appropriate for children with ASD and adapted to the participant’s developmental stage and limitations, it was not effective in addressing the grief. The participant did not show any progress in identifying the emotions connected to the loss and struggled to engage with death-related topics. Additionally, a neuropsychological therapeutic intervention SEEDS (social medicine, education, exercise, diet, and sleep) was adopted to improve the mental health of the grieving participant with ASD. That intervention did not address the grief, but limited progress in general mental health was recognized. The participant did not respond to any treatment targeting grief.

### Environmental Levels of Support Provision

The reported grief support was mainly found at the micro and meso level of the children’s immediate and personal environment, and thus at the individual and family level or at a personal level outside the immediate family. Support was also provided without including the child itself, located at the exo level. No information was given regarding grief support at the macro level. Furthermore, the support components were not evenly distributed, and education about death and dying before the grieving situation was missing altogether (see Table 5).

**Table 5.** Environmental Levels Where Support was Provided.

	Micro Level	Meso Level	Exo Level	Macro Level
Education				
Participation	X	X	X	
Facilitation	X	X	X	
Intervention	X	X		

## Discussion

A variety of support was found in this literature review, including discussions and education about death and grief after losing a beloved one, participation in death rituals and preparation, family support, and professional interventions. Books and stories were helpful resources in facilitating death-related conversations and explaining emotions and coping strategies. However, the results indicate that support components could not be found on all environmental levels (see Table 5). The macro level was missing altogether, as well as education prior to the bereavement. The fact that this level is missing indicates that grief support for children with ID or ASD tends to be irregular and inconsistent, hindering reasonable and sustainable assistance. Read and Elliott (2007) argued in their bereavement support model that continuous comprehensive support could be realized when all components of grief support (education, participation, facilitation, intervention) are applied on four different environmental levels (micro, meso, exo, macro level), not all identified as the case in this review.

### Education Before the Loss

The component education refers to death education before any personal experience of losing a beloved person to prepare for such circumstances (Read & Elliott, 2007). All studies of this literature review were conducted after the death of a beloved person without providing any information about pre-death education. However, education about death, dying, and grief was present and a vital part of both the social and professional support in the included studies. Education about death and dying before any personal experience might be challenging due to many existing reservations and the desire not to put these children in fear (Dodd et al., 2005). Also, the concern that they do not understand the concept of death may prevent this (Read & Elliott, 2007). The included studies presented ways of explaining and educating about death utilizing books and stories, although the education took place after losing someone. However, given the holistic approach by Read and Elliott (2007), the educational topic is missing.

Information regarding the macro level could not be identified. Read and Elliott (2007) emphasized that curriculum development, national initiatives, and funding for research about death education could be located at the macro level. Recognizing the need for education on the macro level is vital for developing guidelines and death-related education programs for children with ID and ASD.

### Children’s Participation in Their Grieving Process

Many of the interventions focused on the micro level, and children with ID or ASD’s participation in grieving processes and rituals are crucial for promoting healthy grief reactions (Dodd et al., 2008). Children with ID or ASD should be encouraged to participate in death rituals to understand and realize that death has occurred (Read & Elliott, 2007). However, actual participation at funerals was only mentioned in two studies focusing on preparation for this event. Two other studies reported that the family decided not to include the child in the death rituals. This finding is consistent with the literature, emphasizing the risk of exclusion from death-related ceremonies, yet they are excluded (Dodd et al., 2005; Sormanti & Ballan, 2011). This review also indicates that these children sometimes are insufficiently informed, and that can, according to Dyregrov (2008), also increase the risk of psychological issues and adverse grief outcomes.

On the meso level, teachers were primarily able to prepare children for death rituals, such as anticipating what would happen and how people might react. Stories and visual aids were additionally used for explanations and preparation, as it is critical to prepare those children so that impressions and other people’s emotions do not overwhelm them (Dyregrov, 2008). We also found that one study deemed participation in religious teaching and praying helpful.

Indirect support on the exo level was another essential aspect of children’s participation. Psychologists in one study advised parents to incorporate their children in death-related rituals and discussions. If the direct caregivers have difficulty dealing with the death, they may need help to include and support the children with ID or ASD (Dyregrov & Dyregrov, 2008). In one included study, psychologists informed parents about the risks of excluding children and how to prepare them best for the event. Advising and supporting parents’ preparation may prevent children from being excluded out of a desire to protect them from strong negative emotions. This underlying desire to protect the children can be harmful when it prevents grieving and might lead to negative long-term consequences (Dodd et al., 2005; Stroebe et al., 2007).

### Facilitation of the Grieving Process

Facilitation in the grieving process includes acknowledging grief and supporting the children in learning to cope with the loss (Read & Elliott, 2007). When children showed or communicated their grief, it facilitated them receiving support. Information about death and dying was considered an essential facilitator throughout the studies, and this information was located close to the child, either within the family or in close proximity outside the family, i.e., micro and meso levels. Families and teachers supported children by discussing death and explaining loss-related feelings. Stories were utilized to educate about death and emotions and present death as a natural life experience. It was emphasized that the stories must be written in clear and direct language. That is consistent with the current literature, where explicit language is necessary to educate children with ID or ASD about death (Hermosilla & Robaina, 2021).

Especially children with ASD need clear information regarding death to understand its consequences (Hume et al., 2016). Wishing to know facts about the circumstances of the death was also found in studies focusing on children with ASD. In another study, religious ideologies and beliefs helped the grieving children cope and conceptualize the idea of death because it was perceived as God’s will and that the deceased was with God. That can be seen as another way of educating children about death because death rituals vary greatly depending on cultural context and beliefs (Haider & Zaman, 2022). Teachers engaged the children in activities like crafts to honor and remember the deceased to facilitate healthy grief reactions. These activities have previously been considered helpful in coping with death (Gilrane-McGarry & Taggart, 2007). Teachers acknowledged the grief and sometimes tried to compensate for the lack of help at the children’s home. They supported parents on the exo level in getting assistance and linking them to other resources that might facilitate children’s grief.

### Professional Grief Intervention

All other components of the model (education, participation, facilitation) aimed to prevent the need for additional professional grief intervention (Read & Elliott, 2007). Three of the six studies included professional intervention, although only two provided information about the intervention process and outcome. Both addressed children with ASD. The family played an essential role in recognizing the need for professional interventions and helping access the intervention; this component was found on the micro and meso levels. No studies about the professional grief intervention process for children with ID could be found in the current literature, indicating a gap in research.

Video games were utilized in one of the studies to facilitate grief therapy and discussions about death and emotions. Using stories that are not directly connected to their feelings helped those children to express emotions. They did not talk about their grief experience but referred to the main characters. Children with ASD tend to have difficulties talking about their feelings (Forrester-Jones & Broadhurst, 2007), and expressing them through a representative character facilitates discussions regarding death and emotions. ‘Playing with death’ in the video game, such as killing or saving other characters, was reported by all participants, allowing them to explore death more comfortably. It might help conceptualize death, a conceptualization considered challenging for children with ASD (Sormanti & Ballan, 2011).

In another study, the intervention targeted complicated grief symptoms, including CGT in the form of cognitive behavioral therapy (CBT) and interventions to improve general mental health. Although the treatment was adapted to the participant with ASD, no improvement in the grieving process could be found. Cognitive behavioral therapy has been considered an appropriate therapy for adults with ASD (Wheeler, 2016), but might differ for children with ASD, where research is lacking. Wheeler (2016) suggested choosing a grief counselor with expertise in ASD, grief interventions, and CBT. Developing professional interventions for children with ID and ASD, such as psychotherapy and accessible grief counseling, requires initiatives on the macro level. The promotion and funding of research are vital to developing evidence-based interventions and guidelines for professionals (Read & Elliott, 2007).

### Limitations of the Study

This review has several limitations. Only a small number of studies were included to answer the research question. That is due to limited research in the field of interest. All studies had a small sample size, and only in two studies did children with ID or ASD express their views on what they perceived as supportive in their grieving process. Both chronological age and cognitive age can impact grief reactions (American Psychiatric Association, 2022), and including children of all ages can be considered a weakness. However, these groups, independent of age, need grief support. Due to the limited research, no generalized assumptions about the grief reactions of children with ID or ASD can be made. This study addressed both children with ID and children with ASD. More research is necessary to get more specific knowledge about grief reactions and grief support targeting each group’s needs. Only two studies could be found that describe professional intervention processes for children with ASD. One had low quality because ethical considerations were not reported, and the second was a PhD thesis. No studies about the professional grief intervention process for children with ID were identified in the current literature.

### Practical Implications and Future Research

Grief support must be adapted to children’s developmental levels and personal needs. Services and involved individuals on all environmental levels must collaborate to ensure comprehensive support. Tools like stories can provide information and support in processing grief. Children with ASD could benefit from supportive stories to explain and explore death and related emotions. However, the literature on grief support is limited. Suitable tools and programs need to be developed and examined for their effectiveness. A research gap was identified in grief support for both children with ID and ASD, whether social or professional.

## Conclusion

This scoping review examined social and professional grief support for children with ID or ASD. Some support has been found in various studies, such as education and discussions about death, preparation, and participation in death rituals, the use of stories about death, and support for the family. Due to the lack of data, no clear statement can be made about the effectiveness of the support, and it cannot be generalized to either of the groups. Children with ID or ASD require support from their environment in coping with grief. However, this can only be adequately ensured if children with ID or ASD are perceived as individuals who are capable of grieving and when their grief needs are acknowledged. Continuous and comprehensive support is recommended to prevent adverse grief outcomes. However, due to the limited data available, no conclusion can be drawn about the feasibility of ongoing comprehensive grief support for children with ID or ASD. More data is vital for developing support programs for children with ID or ASD and identifying potential support methods suitable for these children.
